# Diagnosis Across the Spectrum of Progressive Supranuclear Palsy and Corticobasal Syndrome

**DOI:** 10.1001/jamaneurol.2019.4347

**Published:** 2019-12-20

**Authors:** Edwin Jabbari, Negin Holland, Viorica Chelban, P. Simon Jones, Ruth Lamb, Charlotte Rawlinson, Tong Guo, Alyssa A. Costantini, Manuela M. X. Tan, Amanda J. Heslegrave, Federico Roncaroli, Johannes C. Klein, Olaf Ansorge, Kieren S. J. Allinson, Zane Jaunmuktane, Janice L. Holton, Tamas Revesz, Thomas T. Warner, Andrew J. Lees, Henrik Zetterberg, Lucy L. Russell, Martina Bocchetta, Jonathan D. Rohrer, Nigel M. Williams, Donald G. Grosset, David J. Burn, Nicola Pavese, Alexander Gerhard, Christopher Kobylecki, P. Nigel Leigh, Alistair Church, Michele T. M. Hu, John Woodside, Henry Houlden, James B. Rowe, Huw R. Morris

**Affiliations:** 1 Department of Clinical and Movement Neurosciences, UCL (University College London) Queen Square Institute of Neurology, London, United Kingdom; 2 Movement Disorders Centre, UCL Queen Square Institute of Neurology, London, United Kingdom; 3 Department of Clinical Neurosciences and MRC (Medical Research Council) Cognition and Brain Sciences Unit, University of Cambridge, Cambridge, United Kingdom; 4 Department of Neuromuscular Diseases, UCL Queen Square Institute of Neurology, London, United Kingdom; 5 UK Dementia Research Institute, UCL Queen Square Institute of Neurology, London, United Kingdom; 6 Dementia Research Centre, Department of Neurodegenerative Disease, UCL Queen Square Institute of Neurology, London, United Kingdom; 7 Department of Neurology, Manchester Academic Health Science Centre, Salford Royal NHS (National Health Service) Foundation Trust, University of Manchester, Manchester, United Kingdom; 8 Nuffield Department of Clinical Neurosciences, University of Oxford, Oxford, United Kingdom; 9 Reta Lila Weston Institute, UCL Queen Square Institute of Neurology, London, United Kingdom; 10 Queen Square Brain Bank for Neurological Disorders, UCL Queen Square Institute of Neurology, London, United Kingdom; 11 Department of Psychiatry and Neurochemistry, Institute of Neuroscience and Physiology, Sahlgrenska Academy at the University of Gothenburg, Gothenburg, Sweden; 12 Institute of Psychological Medicine and Clinical Neurosciences, MRC Centre for Neuropsychiatric Genetics and Genomics, Cardiff University, Cardiff, United Kingdom; 13 Institute of Neurological Sciences, Queen Elizabeth University Hospital, Glasgow, United Kingdom; 14 Faculty of Medical Sciences, Newcastle University, Newcastle, United Kingdom; 15 Departments of Geriatrics and Nuclear Medicine, Universitätsklinikum Essen, Essen, Germany; 16 Division of Neuroscience and Experimental Psychology, University of Manchester, Manchester, United Kingdom; 17 Department of Neuroscience, Brighton and Sussex Medical School, Brighton, United Kingdom; 18 Department of Neurology, Royal Gwent Hospital, Newport, United Kingdom

## Abstract

**Importance:**

Atypical parkinsonian syndromes (APS), including progressive supranuclear palsy (PSP), corticobasal syndrome (CBS), and multiple system atrophy (MSA), may be difficult to distinguish in early stages and are often misdiagnosed as Parkinson disease (PD). The diagnostic criteria for PSP have been updated to encompass a range of clinical subtypes but have not been prospectively studied.

**Objective:**

To define the distinguishing features of PSP and CBS subtypes and to assess their usefulness in facilitating early diagnosis and separation from PD.

**Design, Setting, Participants:**

This cohort study recruited patients with APS and PD from movement disorder clinics across the United Kingdom from September 1, 2015, through December 1, 2018. Patients with APS were stratified into the following groups: those with Richardson syndrome (PSP-RS), PSP-subcortical (including PSP-parkinsonism and progressive gait freezing subtypes), PSP-cortical (including PSP-frontal and PSP-CBS overlap subtypes), MSA-parkinsonism, MSA-cerebellar, CBS–Alzheimer disease (CBS-AD), and CBS–non-AD. Data were analyzed from February 1, through May 1, 2019.

**Main Outcomes and Measures:**

Baseline group comparisons used (1) clinical trajectory; (2) cognitive screening scales; (3) serum neurofilament light chain (NF-L) levels; (4) *TRIM11*, *ApoE*, and *MAPT* genotypes; and (5) volumetric magnetic resonance imaging measures.

**Results:**

A total of 222 patients with APS (101 with PSP, 55 with MSA, 40 with CBS, and 26 indeterminate) were recruited (129 [58.1%] male; mean [SD] age at recruitment, 68.3 [8.7] years). Age-matched control participants (n = 76) and patients with PD (n = 1967) were included for comparison. Concordance between the antemortem clinical and pathologic diagnoses was achieved in 12 of 13 patients with PSP and CBS (92.3%) undergoing postmortem evaluation. Applying the Movement Disorder Society PSP diagnostic criteria almost doubled the number of patients diagnosed with PSP from 58 to 101. Forty-nine of 101 patients with reclassified PSP (48.5%) did not have the classic PSP-RS subtype. Patients in the PSP-subcortical group had a longer diagnostic latency and a more benign clinical trajectory than those in PSP-RS and PSP-cortical groups. The PSP-subcortical group was distinguished from PSP-cortical and PSP-RS groups by cortical volumetric magnetic resonance imaging measures (area under the curve [AUC], 0.84-0.89), cognitive profile (AUC, 0.80-0.83), serum NF-L level (AUC, 0.75-0.83), and *TRIM11* rs564309 genotype. Midbrain atrophy was a common feature of all PSP groups. Eight of 17 patients with CBS (47.1%) undergoing cerebrospinal fluid analysis were identified as having the CBS-AD subtype. Patients in the CBS-AD group had a longer diagnostic latency, relatively benign clinical trajectory, greater cognitive impairment, and higher *APOE*-ε4 allele frequency than those in the CBS–non-AD group (AUC, 0.80-0.87; *P* < .05). Serum NF-L levels distinguished PD from all PSP and CBS cases combined (AUC, 0.80; *P* < .05).

**Conclusions and Relevance:**

These findings suggest that studies focusing on the PSP-RS subtype are likely to miss a large number of patients with underlying PSP tau pathology. Analysis of cerebrospinal fluid defined a distinct CBS-AD subtype. The PSP and CBS subtypes have distinct characteristics that may enhance their early diagnosis.

## Key Points

**Questions:**

What are the distinguishing features of progressive supranuclear palsy and corticobasal syndrome subtypes and how can they be distinguished from Parkinson disease?

**Findings:**

In this cohort study of 222 patients with atypical parkinsonian syndromes, recently defined progressive supranuclear palsy subtypes are almost as common as classic Richardson syndrome and share midbrain atrophy as a common hallmark. Distinct patterns of clinical trajectory, cognitive profile, serum neurofilament light chain level, genetic, and volumetric magnetic resonance imaging measures helped to distinguish the clinical subtypes of progressive supranuclear palsy and corticobasal syndrome; clinical trajectory and serum neurofilament light chain levels distinguished Parkinson disease from progressive supranuclear palsy and corticobasal syndrome.

**Meaning:**

This study suggests that subtypes of progressive supranuclear palsy and corticobasal syndrome have distinct characteristics that may enhance their early diagnosis.

## Teaser

This cohort study defines the distinguishing features of progressive supranuclear palsy and corticobasal syndrome and assesses their usefulness in facilitating early diagnosis and separation from Parkinson disease among patients with atypical parkinsonian syndromes.

## Introduction

Atypical parkinsonian syndromes (APS) consist of a heterogeneous group of neurodegenerative disorders that include progressive supranuclear palsy (PSP), corticobasal syndrome (CBS), and multiple system atrophy (MSA). Atypical parkinsonian syndromes are characterized by a more rapid deterioration and poorer levodopa response than is usually seen in Parkinson disease (PD).^1^ In addition, APS are rarer than PD, with an estimated combined prevalence of 10 to 18 per 100 000 population.^2,3,4^ Within APS, there is a high degree of clinical overlap, particularly in early disease, leading to greater misdiagnosis than occurs in PD.^5^ The lack of proven disease-specific diagnostic markers means that postmortem neuropathologic analysis is the criterion standard for confirming the clinical diagnosis.

The recent therapeutic trials of davunetide^6^ and tideglusib^7^ in PSP–Richardson syndrome (PSP-RS) did not result in improved outcomes. The power of clinical trials is limited by individual variability in disease progression and misclassification. Moreover, trials that focus on classic presentations may not be applicable to the full disease spectrum. Accurate diagnosis and prognosis based on clinical and biomarker data may increase statistical power and reduce the required sample size for trials.^8^ The new era of potential disease-modifying therapies for APS has made the need for early and accurate biomarker-supported clinical diagnosis even greater.

The pathologic features of PSP are characterized by 4-repeat tau (4RT) neuronal and glial lesions predominantly in the basal ganglia, brainstem, and cerebellar structures. The classic clinical phenotype of PSP, PSP-RS,^9^ was detailed in the National Institute of Neurological Disorders/Society for PSP (NINDS-SPSP) operational diagnostic criteria of 1996.^10^ Recently, PSP-RS and other non-RS clinical subtypes with underlying PSP pathologic features, such as PSP-parkinsonism and progressive gait freezing (PSP-PGF),^11^ have been incorporated in the Movement Disorder Society (MDS) PSP clinical diagnostic criteria^12^ with independent neuropathologic validation studies showing improved sensitivity compared with the NINDS-SPSP criteria.^13^ These new criteria also recognize the common cognitive presentations of PSP, including changes in behavior and speech and language.

Corticobasal syndrome is a clinical syndrome characterized by progressive asymmetrical limb apraxia, parkinsonism, dystonia, and particular cognitive impairments.^14^ The underlying neuropathologic features of CBS are heterogeneous, with corticobasal degeneration (CBD), PSP, Alzheimer disease (AD), and TAR DNA-binding protein 43 pathologic changes seen at postmortem, even when using the clinical consensus criteria.^15,16^ Multiple system atrophy is an α-synuclein–linked oligodendrogliopathy manifesting with variable combinations of progressive autonomic failure, parkinsonism with poor levodopa response, and cerebellar ataxia.^17^

Herein, we describe the UK-wide Progressive Supranuclear Palsy–Corticobasal Syndrome–Multiple System Atrophy (PROSPECT) study and compare our baseline data with that of patients with PD in the UK-wide Tracking Parkinson’s study to provide a comprehensive prospective picture of the diagnosis and clinical features of PSP and CBS. A strength of the PROSPECT study was the breadth of clinical subtypes that were studied systematically with multiple candidate biomarkers, including indeterminate cases that lay outside of diagnostic criteria when the study was started but came to lie within the current classifications of APS after publication of the MDS PSP critieria.^12^ We examined clinical, cognitive, fluid, genetic, and imaging biomarkers and performed group comparisons, including receiver operating characteristic curves for patient classification.

## Methods

### Study Design

The PROSPECT study natural history cohort consists of 7 UK study sites (University College London [UCL], Oxford, Cambridge, Newcastle, Brighton, Newport, and Manchester). We obtained study-wide ethical approval from the UCL Queen Square Institute of Neurology research ethics committee, recruited participants, and obtained written informed consent from September 1, 2015, through December 1, 2018. We invited all participants to register for postmortem brain donation at 1 of 4 UK brain banks (Queen Square [London], Cambridge, Oxford, and Manchester). Tracking Parkinson’s is a UK-wide longitudinal study of PD. Participants with a baseline clinical diagnosis of PD at 72 sites in the United Kingdom, with multicenter ethics committee and local research and development department approvals, were recruited and provided written informed consent from January 1, 2012, through December 31, 2014.^18^ Postmortem data from patients with PD in the Tracking Parkinson’s study were not available for analysis. This study followed the Strengthening the Reporting of Observational Studies in Epidemiology (STROBE) reporting guideline.

### Participants

We defined patients entering the study as having PSP, following the NINDS-SPSP criteria^10^; CBS, following the Armstrong criteria^14^; or MSA, following the revised Gilman criteria.^17^ We also included patients with progressive movement or cognitive disorders, thought likely to have APS (based on having atypical clinical features for PD) but not meeting any of the above diagnostic criteria, as indeterminate (IDT) cases. Recruited control participants included a spouse or a friend of the case or came through the Join Dementia Research volunteer registry. Cases with PD from the Tracking Parkinson’s study were diagnosed using the Queen Square Brain Bank clinical diagnostic criteria.^19^

### Phenotyping

We reclassified PROSPECT study cases with a diagnosis of PSP, CBS, or IDT according to current MDS PSP criteria^12^ at the end of baseline recruitment.^20^ All reclassified PSP cases fulfilled at least “possible” diagnostic criteria. We stratified PSP cases into PSP-RS, PSP-subcortical, and PSP-cortical groups. The PSP-subcortical group includes cases with PSP-parkinsonism, PSP-PGF, and PSP-oculomotor; the PSP-cortical group includes cases with PSP-CBS overlap and PSP-frontal.

Baseline CBS cases with cerebrospinal fluid (CSF) evidence of underlying AD pathologic features (described in the Fluid Biomarkers subsection below) were defined as CBS-AD, and those with normal CSF analysis were defined as CBD-4RT because they are likely to have underlying CBD or PSP pathologic features. Cases of CBS without CSF or postmortem examination were defined as CBS-unknown. Baseline MSA cases were divided into MSA-parkinsonism and MSA-cerebellar groups according to the revised Gilman criteria.^17^ Cases with PD who have had a change in clinical diagnosis since their baseline Tracking Parkinson’s clinical assessment were excluded from this study.

### Clinical Assessments

We completed core and optional study assessments at baseline. These assessments will be repeated after 6, 12, 24, and 36 months of follow-up, with brief assessments at the 48- and 60-month study visits (eTables 1 and 2 in the Supplement). At each study visit, a neurological history was obtained, and an examination was performed. The PSP Rating Scale^21^ (scores range from 0-100, with higher scores indicating greater impairment) for PSP, CBS, and IDT cases or the Unified Multiple System Atrophy Rating Scale (scores range from 0-104, with higher scores indicating greater impairment)^22^ for MSA cases was administered by a physician. In addition, all cases were assessed using the MDS Unified Parkinson’s Disease Rating Scale parts II (scores range from 0-52, with higher scores indicating greater impairment) and III (scores range from 0-132, with higher scores indicating greater impairment)^23^ and the Schwab and England Activities of Daily Living Scale (SEADL; scores range from 0-100, with lower scores indicating greater impairment).^24^ Cases and controls were screened for cognitive dysfunction using the Montreal Cognitive Assessment (MoCA; scores range from 0-30, with higher scores indicating greater impairment).^25^ Addenbrooke’s Cognitive Examination 3 (ACE-III)^26^ and Edinburgh Cognitive and Behavioural ALS (Amyotrophic Lateral Sclerosis) Screen^27^ were administered as additional, optional cognitive screening assessments. Cases with PD from the Tracking Parkinson’s study underwent baseline testing with the MDS Unified Parkinson’s Disease Rating Scale parts II and III, SEADL, and MoCA.

### Fluid Biomarkers

We measured serum neurofilament light chain (NF-L) levels in a subset of PROSPECT and Tracking Parkinson’s cases, and PROSPECT controls. The CSF total tau (T-tau) and β-amyloid 1-42 (Aβ1-42) levels were measured in a subset of PROSPECT cases at the UK Dementia Research Institute Fluid Biomarker Laboratory at UCL (eMethods in the Supplement). Cases with CBS were stratified into groups with likely underlying AD pathologic features (CBS-AD), defined as cases with a CSF T-tau:Aβ1-42 ratio of greater than 1^28^; likely 4RT pathologic features (CBS-4RT), defined as cases with a CSF T-tau:Aβ1-42 ratio of less than 1; and unknown pathologic features (CBS-unknown), defined as cases with no CSF analysis.

### Genetics

A subset of PROSPECT and Tracking Parkinson cases had DNA extracted from blood samples. DNA was subsequently used for genotyping and single-nucleotide polymorphism imputation (eMethods in the Supplement) to obtain *MAPT* (OMIM 157140) H1/H1, *APOE* (OMIM 107741) ε4 allele, and *TRIM11* (OMIM 607868) rs564309 minor allele group frequencies.

### Neuroimaging

A subset of PROSPECT participants attended 3 scanning centers (UCL, Cambridge, and Oxford) and underwent baseline volumetric T1-weighted magnetic resonance imaging (MRI) on 3T scanners (Siemens, Prisma, or TRIO) (eMethods in the Supplement). We combined the basal ganglia (caudate, putamen, and pallidum), accumbens, and thalamus as central structures for summarizing groupwise subcortical atrophy. Imaging data from Tracking Parkinson’s participants were not available.

### MSA Group Data

We have included cases with MSA in the description of our PROSPECT study cohort and baseline clinical features. However, the statistical analyses described below and comparisons with PD data have been restricted to PSP, CBS, and IDT cases because these cases were reclassified under the MDS PSP diagnostic criteria. The analysis of associated MSA group data will be published separately.

### Statistical Analysis

Data were analyzed from February 1 through May 1, 2019, using Plink, version 1.9 (Harvard University), GraphPad, version 8 (Prism), and Stata, version 15 (StataCorp LLC). For missing data in clinical scales, an adjusted mean score was used if at least 80% of the assessment was complete.

Group comparisons of clinical, cognitive, and biomarker measures were made using logistic regression analyses with sex, age at symptom onset, and disease duration at testing as covariates. We calculated the clinical disease trajectory by dividing PSP Rating Scale and MDS Unified Parkinson’s Disease Rating Scale parts II and III scores at baseline by the number of years since reported motor symptom onset, assuming a score of 0 immediately before symptom onset. For the SEADL, the clinical disease trajectory was calculated as (100 − baseline score) divided by the number of years since reported motor symptom onset, assuming a score of 100 immediately before symptom onset. Statistical significance for the clinical, cognitive, and biomarker group comparisons described above was defined as a false discovery rate–corrected, 2-sided *P* < .05. Group comparisons of genetic data were made using Fisher exact tests, and statistical significance was defined as a Bonferroni-corrected 2-sided *P* < .05. We performed an analysis of covariance on imaging volumetric measures from each brain region, with diagnosis and sex as factors and age at scan and total intracranial volume as covariates. Regional marginal mean values were compared post hoc using unpaired *t* tests. The significance of mean differences was adjusted using false discovery rate correction with 2-sided *P* < .05 considered significant. In addition, receiver operating characteristic curve analyses were performed on cognitive scale, serum NF-L level, and regional imaging volumetric values from group pairs with the area under the curve (AUC) used as a measure of separation between the groups.

## Results

### Recruitment and Phenotyping

**Figure 1.**
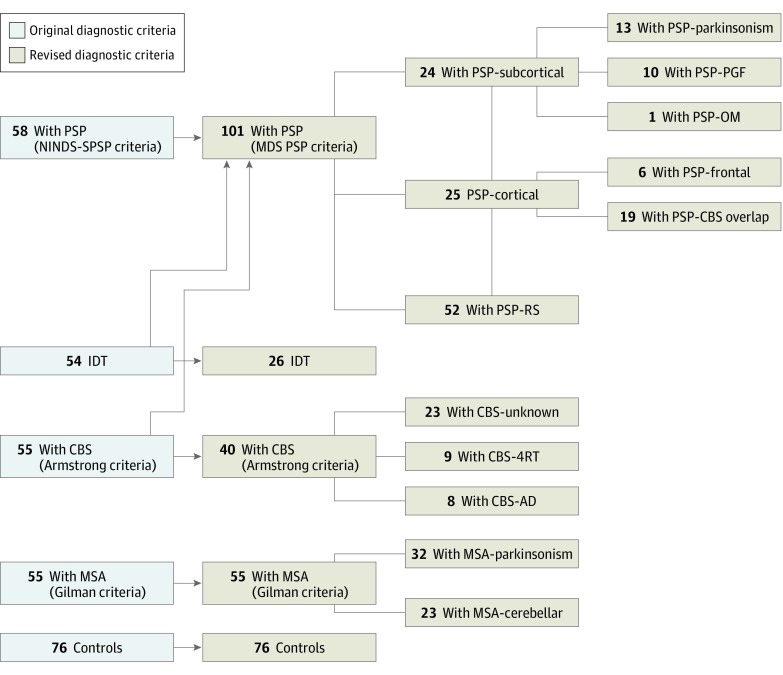
Recruitment of Patients to the Progressive Supranuclear Palsy–Corticobasal Syndrome–Multiple System Atrophy (PROSPECT) Study AD indicates Alzheimer disease; CBS, corticobasal syndrome; 4RT, 4-repeat tau; IDT, indeterminate; MDS, Movement Disorder Society; MSA, multiple system atrophy; NINDS-SPSP, National Institute of Neurological Disorders/Society for PSP (Progressive Supranuclear Palsy); OM, oculomotor; PGF, progressive gait freezing; and RS, Richardson syndrome.

We analyzed 222 cases with APS (93 female [41.9%] and 129 male [58.1%]; mean [SD] age at recruitment, 68.3 [8.7] years), 76 controls, and 1967 cases with PD. At study entry, application of clinical diagnostic criteria, including the NINDS-SPSP PSP criteria, identified 58 cases with PSP, 55 cases with MSA, 55 cases with CBS, and 54 IDT cases (Figure 1). Reclassification of PROSPECT PSP, CBS, and IDT cases was possible after the publication of the 2017 MDS PSP criteria, resulting in 101 cases with PSP, 55 cases with MSA, 40 cases with CBS, and 26 IDT cases (Figure 1). Of note, 15 cases with CBS were reclassified as PSP-CBS overlap under the MDS PSP diagnostic criteria because they had the presence of slowed vertical saccades and/or a vertical supranuclear gaze palsy, both of which are associated with underlying PSP pathologic findings.^1^ In total, 17 of 40 reclassified CBS cases (42.5%) had CSF collection, of whom 8 (47.1%) had an AD-like CSF profile. The following disease groups were defined (Figure 1): PSP-RS, PSP-subcortical (consisting of PSP-parkinsonism, PSP-PGF, and PSP–oculomotor predominant subtypes), PSP-cortical (consisting of PSP-CBS overlap and PSP-frontal subtypes), MSA-parkinsonism, MSA-cerebellar, CBS-unknown, CBS-4RT, CBS-AD, and IDT.

### Pathologic Confirmation of Diagnosis

Forty-four of 222 cases in the PROSPECT cohort (19.8%) had died at the point of censoring, with a mean (SD) disease duration of 5.9 (2.3) years. Seventeen of 44 cases (38.6%) had pathologic confirmation of diagnosis at the Queen Square, Cambridge, Oxford, and Manchester brain banks with concordance between antemortem clinical and pathologic diagnoses achieved in 12 of 13 cases with PSP and CBS (92.3%) (Table 1). Of note, the clinically diagnosed case with PSP-RS who had CBD pathologic findings at postmortem had typical features of PSP-RS with no evidence of apraxia throughout the disease course and was therefore classified as a case of PSP-RS under the NINDS-SPSP and MDS diagnostic criteria.

**Table 1.** Baseline Clinical Features of the PROSPECT Study Natural History Cohort^a^

Characteristic	Study Group													
	Controls (n = 76)	PSP by Subgroup				MSA by Subgroup			CBS by Subgroup				IDT (n = 26)	PD (n = 1763)^b^
		All (n = 101)	RS (n = 52)	Subcortical (n = 24)	Cortical (n = 25)	All (n = 55)	Parkinsonism (n = 32)	Cerebellar (n = 23)	All (n = 40)	Unknown (n = 23)	4RT (n = 9)	AD (n = 8)		
Sex, No. (%) male	28 (36.8)	63 (62.4)	33 (63.5)	18 (75.0)	12 (48.0)	36 (65.5)	17 (53.1)	19 (82.6)	14 (35.0)	7 (30.4)	4 (44.4)	3 (37.5)	16 (61.5)	1146 (65.0)
Age at enrollment, y	66.7 (8.4)	70.6 (7.3)	69.2 (7.8)	73.1 (6.0)	71.1 (7.0)	63.6 (10.1)	63.7 (10.4)	63.5 (9.9)	68.4 (7.4)	68.3 (6.8)	67.5 (8.6)	69.7 (8.3)	69.2 (9.3)	67.3 (9.5)
Age at motor symptom onset, y	NA	66.3 (7.4)	65.9 (7.3)	66.5 (8.2)	66.9 (7.2)	58.1 (10.8)^c^	58.5 (11.3)	57.5 (10.1)	63.6 (7.2)	63.9 (7.6)	61.8 (9.6)	64.6 (7.6)	65.2 (9.1)	64.4 (9.7)
Disease duration at enrollment, y	NA	4.4 (2.7)	3.4 (2.2)	6.6 (3.7)^d^	4.3 (2.2)	5.4 (2.8)	5.2 (2.8)	5.9 (2.7)	4.9 (3.2)	4.3 (3.0)	5.8 (3.7)	5.2 (3.3)	4.1 (2.9)	3.0 (3.1)^e^
Diagnostic latency, y	NA	2.8 (2.2)	2.3 (1.8)	4.2 (3.2)^d^	2.7 (1.3)	3.2 (2.5)	2.8 (2.2)	3.6 (2.8)	3.2 (3.0)	2.8 (2.0)	3.1 (4.7)	4.6 (3.2)	NA	1.8 (2.8)^e^
Baseline score														
PSPRS^f^	NA	34.6 (14.5)	35.7 (15.1)	27.8 (12.0)^d^	39.1 (13.5)	NA	NA	NA	33.5 (17.9)	32.3 (17.3)	41.3 (15.6)	28.4 (21.4)	22.9 (13.2)	NA
SEADL^g^	NA	54.5 (17.6)	53.3 (18.1)	63.2 (16.3)^d^	48.8 (15.0)	57.1 (15.7)	55.6 (14.1)	59.2 (17.7)	54.3 (19.1)	54.5 (20.3)	50.1 (19.8)	58.4 (15.4)	70.4 (17.7)	88.1 (12.0)^e^
No. deceased	NA	23	9	7	7	10	5	5	8	3	4	1	3	NA
No. with postmortem diagnosis	NA	PSP in 9 and CBD in 1	PSP in 4 and CBD in 1	PSP in 3	PSP in 2	MSA in 3 and PD in 1	MSA in 1 and PD in 1	MSA in 2	CBD in 3	CBD in 1	CBD in 2	0	0	NA

Abbreviations: AD, Alzheimer disease; CBD, corticobasal degeneration; CBS, corticobasal syndrome; CSF, cerebrospinal fluid; 4RT, 4-repeat tau; IDT, indeterminate; MSA, multiple system atrophy; NA, not applicable; PD, Parkinson disease; PROSPECT, Progressive Supranuclear Palsy–Corticobasal Syndrome–Multiple System Atrophy; PSP, progressive supranuclear palsy; PSPRS, PSP Rating Scale; RS, Richardson syndrome; SEADL, Schwab and England Activities of Daily Living Scale.

^a^ Indicates baseline clinical features of the PROSPECT study natural history cohort, defined by patients’ reclassified baseline diagnoses. Group and subgroup comparisons used unpaired *t* testing. Unless otherwise indicated, data are expressed as mean (SD).

^b^ Data are from the Tracking Parkinson’s study.

^c^ False discovery rate (FDR) adjusted *P* < .05 vs PSP-all.

^d^ FDR adjusted *P* < .05 vs PSP-RS and PSP-cortical.

^e^ FDR adjusted *P* < .05 vs PSP-all, CBS-all, and MSA-all.

^f^ Scores range from 0 to 100, with higher scores indicating greater impairment.

^g^ Scores range from 0 to 100, with lower scores indicating greater impairment.

### Clinical Features

All PROSPECT cases underwent baseline clinical testing, whereas baseline clinical data were obtained from 1763 of 1967 recruited patients with PD (89.6%) in the Tracking Parkinson’s study. Thirty-two of 204 patients with PD (15.7%) were excluded from this analysis owing to missing data or a change in diagnosis at the point of data analysis.

**Figure 2.**
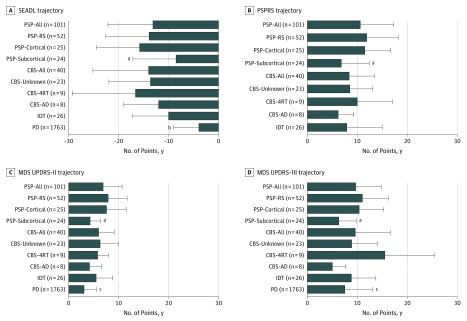
Clinical Disease Trajectory Profiles Data are expressed as mean (SD [error bars]). Group comparisons are adjusted for sex and age at symptom onset. AD indicates Alzheimer disease; CBS, corticobasal syndrome; 4RT, 4-repeat tau; IDT, indeterminate; MDS UPDRS-II, Movement Disorder Society Unified Parkinson’s Disease Rating Scale part II; MDS UPDRS-III, MDS UPDRS part III; PD, Parkinson disease; PSPRS, PSP (Progressive Supranuclear Palsy) Rating Scale; RS, Richardson syndrome; and SEADL, Schwab and England Activities of Daily Living Scale. ^a^False discovery rate (FDR)–adjusted *P* < .05, PSP-subcortical vs PSP-RS and PSP-cortical. ^b^FDR-adjusted *P* < .01, PD vs PSP-all and CBS-all. ^c^FDR-adjusted *P* < .05, PD vs PSP-all and CBS-all.

The baseline clinical features of reclassified cases and controls are summarized in Table 1. There was a long diagnostic delay for CBS-AD (mean [SD], 4.6 [3.2] years) and PSP-subcortical (mean [SD], 4.2 [3.2] years) groups compared with the CBS-4RT (mean [SD], 3.1 [4.7] years) and PSP-RS (mean [SD], 2.3 [1.8] years) groups. There was also variation in the burden of disease at study enrollment as measured by the baseline PSP Rating Scale and SEADL scores, with the highest degree of impairment seen in the PSP-RS (mean [SD] scores, 35.7 [15.1] and 53.3 [18.1], respectively), PSP-cortical (mean [SD] scores, 39.1 [13.5] and 48.8 [15.0], respectively), and CBS-4RT (mean [SD] scores, 41.3 [15.6] and 50.1 [19.8], respectively) groups (Table 1). Clinical trajectory analyses (Figure 2), in particular the SEADL, showed that the PSP-subcortical (mean [SD] decline in score, −8.5 [8.6] points per year), CBS-AD (mean [SD] decline in score, −12.0 [7.0] points per year), and PD (mean [SD] decline in score, −3.9 [5.1] points per year) groups had more benign disease trajectories than all other groups.

### Cognitive Profiles

We evaluated cognitive function using the MoCA, Edinburgh Cognitive and Behavioural ALS Screen, and ACE-III. Among the PROSPECT cases and controls, the MoCA was completed in 235 of 243 participants (96.7%); the Edinburgh Cognitive and Behavioural ALS screen, in 211 of 243 (86.8%); and the ACE-III, in 223 of 243 (91.8%) (eTable 3 in the Supplement). The 3 assessments were strongly correlated (all comparisons, *r* > 0.80). Among the 1967 patients with PD in the Tracking Parkinson’s study, 1833 (93.2%) had baseline MoCA testing (eTable 3 in the Supplement). With regard to total scores, the PD group had better cognition (mean [SD] score, 24.9 [3.6]) compared with the PSP-all (mean [SD] score, 21.9 [4.7]) and CBS-all (20.4 [7.4]) groups. The PSP-cortical group was more impaired across all 3 scales compared with the PSP-RS and PSP-subcortical groups (false discovery rate corrected, *P* < .05). The CBS-AD group had worse cognition in all scales compared with CBS-4RT, but the statistical comparisons were likely limited by small group sizes, with significance reached only in MoCA total score (mean [SD] score, 22.9 [5.3] for CBS-4RT and 12.4 [9.0] for CBS-AD) and ACE-III attention, memory, and language subscale measures (eTable 3 in the Supplement).

### Fluid Biomarkers

Testing of serum samples for NF-L levels was performed in 186 of 243 PROSPECT cases and controls (76.5%) and 140 of 1967 PD cases (7.1%) in the Tracking Parkinson’s study. Forty-four of 167 cases (26.3%) in the PROSPECT study had CSF testing for T-tau and Aβ1-42 levels.

**Figure 3.**
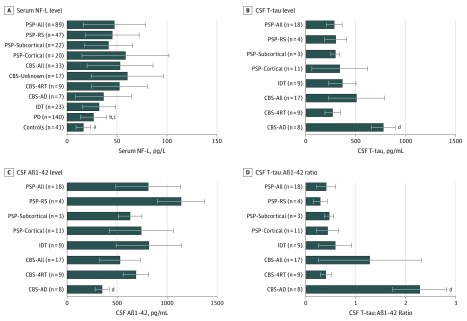
Fluid Biomarker Profiles Data are expressed as mean (SD [error bars]). Group comparisons are adjusted for sex, age at symptom onset, and disease duration at testing. Aβ1-42 indicates β-amyloid 1-42; AD, Alzheimer disease; CBS, corticobasal syndrome; CSF, cerebrospinal fluid; 4RT, 4-repeat tau; IDT, indeterminate; NF-L, neurofilament light chain; PD, Parkinson disease; PSP, progressive supranuclear palsy; RS, Richardson syndrome; and T-tau, total tau. ^a^False discovery rate (FDR)–adjusted *P* < .01, controls vs all disease groups. ^b^FDR-adjusted *P* < .05, PD vs PSP-all. ^c^FDR-adjusted *P* < .05, PD vs CBS-all. ^d^FDR-adjusted *P* < .01, CBS-AD vs all other disease groups.

At the group level, serum NF-L levels in patients with PD (26.5 pg/L) were significantly higher than in controls (16.4 pg/L) and the PSP-all (47.4 pg/L) and CBS-all (53.1 pg/L) groups. Serum NF-L levels did not distinguish between the PSP-all and CBS-all groups (Figure 3). With respect to disease subgroups, there was a trend toward higher mean serum NF-L levels in PSP-cortical (58.6 pg/L) vs PSP-RS (45.3 pg/L) and PSP-subcortical (41.6 pg/L) and in CBS-4RT (52.4 pg/L) vs CBS-AD (36.5 pg/L) (Figure 3).

### Genetics

Genotype data were obtained from 134 of 167 PROSPECT cases (80.2%) and 1566 of 1967 PD cases (79.6%) in the Tracking Parkinson’s study (eTable 4 in the Supplement). In the analysis of white cases only, we found significantly higher *MAPT* H1/H1 frequencies in the PSP-all (88.9%) and CBS-all (78.8%) groups compared with the PD group (67.2%) and reference controls (67.1%) (Bonferroni-corrected *P* < .05). At the subgroup level, we found significantly higher *APOE*-ε4 allele frequencies in CBS-AD (35.7%) compared with CBS-4RT (Bonferroni-corrected *P* < .05). Although analyses were underpowered to reach significance, as reported previously,^29^ we found higher *TRIM11* rs564309 minor allele frequencies in PSP-subcortical (15.0%) compared with PSP-RS (7.1%).

### Magnetic Resonance Imaging

Volumetric measures from T1-weighted MRI scans were derived for 108 of 243 PROSPECT cases and controls (44.4%). eTable 5 in the Supplement outlines the differences in cortical and subcortical volumetric measures across all groups, with post hoc pairwise group comparisons of each patient group vs controls and selected comparisons between patient groups (Table 2). Midbrain atrophy was a consistent neuroimaging feature in all PSP groups (marginal mean [SD] volume: 5.99 [0.53] mL in controls; 5.01 [0.54] mL in PSP-RS; 5.23 [0.54] mL in PSP-subcortical; and 5.16 [0.55] mL in PSP-cortical). However, there was a dissociation between subcortical and cortical variants of PSP: the PSP-subcortical group showed less atrophy in the midbrain, medulla, and central structures, with relatively preserved cortical volumes; the PSP-cortical group showed additional severe frontotemporal atrophy. Corticobasal syndromes were associated with relative preservation of the pons and midbrain (marginal mean [SD] volume: 14.72 [1.70] mL and 5.99 [0.53] mL, respectively, in controls; 13.67 [1.68] mL and 5.54 [0.52] mL, respectively, in CBS-all) but severe atrophy of the central structures and cerebral cortex. Atrophy varied according to whether the CSF AD biomarkers were positive or not, with especially prominent ventriculomegaly in cases with CBS-AD (marginal mean [SD] volume: 35.80 [19.25] mL in controls; 60.81 [18.83] mL in CBS-4RT; 75.75 [18.81] mL in CBS-AD). The IDT cases were notable for their preserved posterior fossa structures, with atrophy of central structures and cerebral cortex.

**Table 2.** Cognitive, Fluid Biomarker, and Imaging AUC Values From ROC Curve Analyses

Variable^a^	AUC by Group Comparison						
	Controls vs PSP-All	Controls vs CBS-All	PSP-All vs CBS-All	PSP-RS vs PSP-Cortical	PSP-RS vs PSP-Subcortical	PSP-Cortical vs PSP-Subcortical	CBS-AD vs CBS-4RT
MoCA	0.84	0.79	0.66	0.75	0.83	0.80	0.87
ECAS	0.91	0.83	0.61	0.73	0.82	0.80	0.80
ACE-III	0.94	0.88	0.64	0.76	0.81	0.81	0.80
Serum NF-L	0.88	0.91	0.72	0.74	0.83	0.75	0.76
Pons-midbrain ratio	0.69	0.73	0.84	0.71	0.58	0.60	0.66
Imaging region							
Pons	0.76	0.92	0.78	0.59	0.58	0.70	0.57
Midbrain	0.89	0.91	0.56	0.63	0.65	0.60	0.60
Medulla	0.78	0.82	0.51	0.54	0.59	0.52	0.69
Cerebellum	0.59	0.79	0.55	0.73	0.54	0.80	0.63
Frontal lobe	0.80	0.71	0.75	0.76	0.70	0.89	0.57
Parietal lobe	0.69	0.60	0.73	0.73	0.60	0.84	0.69
Temporal lobe	0.79	0.67	0.77	0.65	0.60	0.75	0.51
Occipital lobe	0.59	0.63	0.61	0.72	0.52	0.78	0.54
Central structures	0.88	0.81	0.74	0.62	0.50	0.64	0.57
Ventricles	0.71	0.51	0.81	0.60	0.68	0.79	0.66

Abbreviations: ACE-III, Addenbrooke’s Cognitive Examination 3; AD, Alzheimer disease; AUC, area under the ROC curve; CBS, corticobasal syndrome; ECAS, Edinburgh Cognitive and Behavioural ALS (Amytrophic Lateral Sclerosis) Screen; 4RT, 4-repeat tau; MoCA, Montreal Cognitive Assessment; NF-L, neurofilament light chain; PSP, progressive supranuclear palsy; ROC, receiver operating characteristic; RS, Richardson syndrome.

^a^ Cognitive scale and serum NF-L AUC values are based on logistic regression analyses that used sex, age at symptom onset, and disease duration at testing as covariates. Imaging group comparisons were adjusted for sex, age at scan, and total intracranial volume. An AUC of 0.80 or greater indicated high diagnostic accuracy.

### Receiver Operating Characteristic Curve Analyses

Receiver operating characteristic curve analyses showed that PD was distinguished from an APS group, which consisted of all PSP and CBS cases, using serum NF-L levels (AUC, 0.80) (eFigure in the Supplement) and the MoCA score (AUC, 0.78). In addition, we highlight measures that had high diagnostic accuracy (defined by an AUC ≥ 0.80) in differentiating between subgroups (Table 2). All cognitive measures (MoCA, Edinburgh Cognitive and Behavioural ALS Screen, and ACE-III) differentiated CBS-AD from CBS-4RT (AUC, 0.80-0.87) and PSP-subcortical from PSP-RS and PSP-cortical (AUC, 0.80-0.83). In addition, PSP-subcortical was distinguished from PSP-RS using serum NF-L levels (AUC, 0.83) and from PSP-cortical using cortical volumetric MRI measures (AUC, 0.80-0.89).

## Discussion

In this cohort study, we assessed a large number of patients with PSP and CBS recruited to the natural history arm of the PROSPECT study. Although this is not a community-based epidemiologic study, we recruited patients with APS across the United Kingdom. We characterized the different clinical presentations of PSP using the new MDS PSP diagnostic criteria. We identified disease- and subtype-specific markers that are likely to improve the early and accurate differentiation of PD from PSP and CBS and increase the power of future clinical trials with more homogeneous disease groups. We believe these findings should have a direct effect on the new era of anti-tau clinical trials that aim to recruit patients with early-stage PSP.^30^

Our PSP-RS group data are consistent with data from patients with PSP-RS in the davunetide^6^ and tideglusib^7^ trials with regard to clinical (age at symptom onset and recruitment), genetic, and imaging profiles and the degree of motor and/or functional impairment at study recruitment. In addition, our baseline PSP Rating Scale and SEADL scores for patients with PSP-RS were consistent with those seen in patients with PSP-RS recruited to the 4RT neuroimaging initiative longitudinal cohort.^31^

Use of the 2017 MDS PSP criteria increased the number of clinical PSP cases from 58 to 101, implying that non–Richardson syndrome presentations (49 of 101 [48.5%]) are common. The subcortical presentations of PSP, consisting of PSP-parkinsonism and PSP-PGF phenotypes, have a long delay to diagnosis that can at least in part be attributed to frequent initial misdiagnoses as PD, because they share similar clinical trajectories and initial clinical features. Although the present study was not adequately powered to detect significant differences between PSP and CBS subgroups and PD, we were able to detect trends of greater cognitive impairment and higher levels of serum NF-L in the PSP-subcortical group compared with PD.

The PSP-subcortical group had a more benign clinical trajectory, less cognitive impairment, lower serum NF-L levels, higher *TRIM11* rs564309 minor allele frequency, and more restricted midbrain and cortical atrophy than the PSP-RS and PSP-cortical groups. However, we identified midbrain atrophy to be a core neuroimaging feature of PSP across the different subtypes, which may enable early separation from Lewy body PD. The finding of higher serum NF-L levels in the PSP-RS and PSP-cortical groups may indicate higher disease intensity or be a consequence of greater cortical atrophy seen in these phenotypes.

Pathologically proven CBS-AD and CBS-CBD may be difficult to distinguish in clinical practice.^15,16^ The advent of AD biomarkers is likely to improve this differentiation. We found that a biomarker-defined CBS-AD group had a milder clinical trajectory, greater ventriculomegaly, higher *APOE*-ε4 allele frequency, and greater cognitive impairment compared with the CBS-4RT group. In particular, the ACE-III revealed significant differences in attention, memory, and language subscores between CBS-AD and CBS-4RT. Our data show that despite the clinical overlap, CBS-AD can be distinguished from CBS-4RT in life. This finding is further supported by the fact that both of our CSF biomarker-defined CBS-4RT cases with postmortem evaluation had CBD pathologic findings. Although all the major syndromes studied herein are bilateral brain diseases, CBS is typically asymmetrical, in contrast to PSP-RS and MSA. Such asymmetry can be quantified by a laterality index of motor features, but less so in terms of cognitive asymmetry. We therefore opted for a simple general linear model for MRI analysis without laterality. Further increases in the diagnostic accuracy of MRI may be gained in future studies by incorporating phenotypic data, including laterality effects, in the model.

To compare the discriminant usefulness of multimodal biomarkers, accommodating widely different scales and variances, we have also presented their performance as AUC values for cases vs controls and comparisons of disease groups. Using an AUC cutoff of at least 0.80 to represent high diagnostic accuracy, we confirmed the role of cognitive screening scales in differentiating CBS-AD from CBS-4RT and the role of cognitive screening scales, serum NF-L levels, and cortical volumetric MRI measures in differentiating PSP-subcortical from PSP-RS and PSP-cortical. In addition, serum NF-L level (AUC, 0.80) was able to accurately distinguish PD from a combined APS group consisting of all PSP and CBS cases. The comparison of Table 2 and eTables 3 and 5 in the Supplement highlights that the utility of a biomarker to discriminate patient groups (such as the AUC) cannot simply be inferred from the significance of an unpaired *t* test between groups, especially where group sizes vary.

The 2017 MDS PSP diagnostic criteria were published during study recruitment, and so we are able to report the prospective characterization of variant PSP phenotypes using clinical, cognitive, fluid biomarker, genetic, and imaging measures with neuropathologic confirmation of diagnosis. In addition, we present a biomarker-defined CBS-AD group that has distinct clinical, cognitive, and genetic features that allow it to be distinguished from CBS-4RT. We found that as many as 50% of cases with PSP presented with nonclassic variant PSP phenotypes, and in retrospective case series, this frequency has been shown to be as high as 76%.^32^ Until now, these PSP variants have been missed by clinical, therapeutic, and epidemiologic studies that have largely focused on the classic PSP-RS presentation.^2,3,4,6,7^ Similarly, as many as 50% of CBS cases with CSF analysis had a biomarker profile consistent with underlying AD pathologic features. Of note, our estimates are higher than those seen in a similar-sized retrospective case series with pathologic confirmation in which 5 of 21 CBS cases (23.8%) had primary pathologic AD findings at postmortem.^15^ Alongside these phenotype-specific markers, our inclusion of data from a large PD cohort allowed us to confirm that the use of serum NF-L levels and cognitive screening scales may aid the early differentiation of PD from APS.^33,34^

### Limitations

We acknowledge limitations to the present study. First, most of our cases were diagnosed using clinical criteria without neuropathologic verification. Cases with CBS-4RT and CBS-AD were defined using CSF biomarker criteria, and we acknowledge that in late life, AD biomarker positivity may be coincidental alongside CBD and does not prove that AD pathologic features are the primary cause of the clinical symptoms. We anticipate that follow-up of this natural history cohort, with further cases undergoing postmortem assessment, will allow us to validate the sensitivity and specificity of the clinical and biomarker criteria used to stratify patients. Although not currently available in our cohort, in-depth pathologic characterization of APS subtypes and associations with their antemortem biomarker profiles are informative. Previously, pathologic variants of PSP have been described.^35^ Of interest, that study found a higher density of cortical tau pathology in variants of PSP presenting with focal cortical syndromes compared with PSP-parkinsonism and PSP-PGF, a finding that is in line with differences in cortical atrophy seen in PSP-cortical vs PSP-subcortical groups in our study. Our clinical disease trajectory analyses were based on baseline clinical and functional rating scale scores. We believe longitudinal data from this cohort will be essential to accurately characterize the clinical progression of PSP and CBS and identify markers that predict and track progression. Although our AUC results are promising, as we gather more longitudinal data, we expect the diagnostic accuracy of PSP and CBS to further improve with a well-powered multivariate approach, including cross-validated machine learning algorithms. Although a proportion of our IDT cases will eventually have non-APS diagnoses such as PD and vascular gait disorders, we expect that some cases will eventually fulfill diagnostic criteria for defined APS, representing cases that have been recruited at the very earliest disease stages.

## Conclusions

The PROSPECT study’s multimodal assessment of clinical, cognitive, fluid, genetic, and imaging data has identified markers that enable the differentiation of PD from PSP and CBS. In addition, we present confirmatory data on the changes across modalities in classical phenotypes of PSP and CBS and evaluate biomarkers of variant PSP syndromes included in the most recent diagnostic criteria and in a distinct biomarker-defined CBS-AD syndrome. These findings may enhance the early diagnosis of PSP and CBS for accurate prognostication and stratification of patients for clinical trials.

## Notes

Supplement. **eTable 1.** Overview of Planned Assessments in the PROSPECT Study Natural History Cohort **eTable 2.** Clinical Rating Scales, Functional Rating Scales, and Cognitive Screening Tests Used in the PROSPECT Study Natural History Cohort **eTable 3.** Cognitive Screening Measures in the PROSPECT Study Natural History Cohort **eTable 4.** Genetic Analyses in the PROSPECT Study Natural History Cohort **eTable 5.** Volumetric MRI Measures in the PROSPECT Study Natural History Cohort **eMethods.** Fluid Biomarkers, Genetics, and Neuroimaging **eFigure.** Serum NF-L ROC Curve for APS vs PD **eReferences.**
