# Supplementary material for: Diagnosis Across the Spectrum of Progressive Supranuclear Palsy and Corticobasal Syndrome
Source: JAMA Neurol. 2019 Dec 20;77(3). doi: 10.1001/jamaneurol.2019.4347 (PMC6990759; doi:10.1001/jamaneurol.2019.4347)
Supplement: Supplement. — eTable 1. Overview of Planned Assessments in the PROSPECT Study Natural History Cohort eTable 2. Clinical Rating Scales, Functional Rating Scales, and Cognitive Screening Tests Used in the PROSPECT Study Natural History Cohort eTable 3. Cognitive Screening Measures in the PROSPECT Study Natural History Cohort eTable 4. Genetic Analyses in the PROSPECT Study Natural History Cohort eTable 5. Volumetric MRI Measures in the PROSPECT Study Natural History Cohort eMethods. Fluid Biomarkers, Genetics, and Neuroimaging eFigure. Serum NF-L ROC Curve for APS vs PD eReferences. [file jamaneurol-77-377-s001.pdf]

Supplementary Online Content

Jabbari E, Holland N, Chelban V, et al. Diagnosis across the spectrum of progressive supranuclear palsy and corticobasal syndrome. *JAMA Neurol.* Published online December 20, 2019. doi:10.1001/jamaneurol.2019.4347

eTable 1. Overview of Planned Assessments in the PROSPECT Study Natural History Cohort

eTable 2. Clinical Rating Scales, Functional Rating Scales, and Cognitive Screening Tests Used in the PROSPECT Study Natural History Cohort

eTable 3. Cognitive Screening Measures in the PROSPECT Study Natural History Cohort

eTable 4. Genetic Analyses in the PROSPECT Study Natural History Cohort

eTable 5. Volumetric MRI Measures in the PROSPECT Study Natural History Cohort

eMethods. Fluid Biomarkers, Genetics, and Neuroimaging

eFigure. Serum NF-L ROC Curve for APS vs PD

eReferences.

This supplementary material has been provided by the authors to give readers additional information about their work.

© 2019 Jabbari E et al. *JAMA Neurology*.

eTable 1. Overview of Planned Assessments in the PROSPECT Study Natural History Cohort

	Study visit						
	0m	6m	12m	24m	36m	48m	60m
**Core study assessments**							
Clinical rating scales	✓	✓	✓	✓	✓	✓	✓
Functional rating scales	✓	✓	✓	✓	✓	✓	✓
Cognitive screening tests	✓	✓	✓	✓	✓		
Neuropsychometric tests	✓	✓	✓	✓	✓		
Blood sample for biomarkers (and genetics at 0m only)	✓	✓	✓	✓	✓		
Smell test	✓						
**Optional biomarker assessments**							
Skin biopsy	✓		✓				
Magnetic resonance imaging	✓		✓				
Cerebrospinal fluid collection	✓	✓	✓				

eTable 2. Clinical Rating Scales, Functional Rating Scales, and Cognitive Screening Tests Used in the PROSPECT Study Natural History Cohort

	Study visit						
	0m	6m	12m	24m	36m	48m	60m
**Clinician assessed**							
PSPRS/UMSARS	✓	✓	✓	✓	✓	✓	✓
MDS UPDRS-III	✓	✓	✓	✓	✓	✓	✓
SEADL	✓	✓	✓	✓	✓	✓	✓
MoCA	✓	✓	✓	✓	✓		
ECAS	✓	✓	✓	✓	✓		
ACE-III	✓	✓	✓	✓	✓		
**Patient self-assessed**							
MDS UPDRS-II	✓	✓	✓	✓	✓	✓	✓

Addenbrooke's cognitive examination 3: ACE-III, Edinburgh cognitive and behavioural ALS screen: ECAS, Montreal cognitive assessment: MoCA, Schwab and England activities of daily living scale: SEADL, PSP rating scale: PSPRS, Unified Multiple System Atrophy rating scale: UMSARS, Movement Disorder Society Unified Parkinson's Disease Rating Scale part II: MDS UPDRS-II, Movement Disorder Society Unified Parkinson's Disease Rating Scale part III: MDS UPDRS-III.

© 2019 Jabbari E et al. *JAMA Neurology*.

eTable 3. Cognitive Screening Measures in the PROSPECT Study Natural History Cohort

	Controls	PSP-All	PSP-RS	PSP-Subcortical	PSP-Cortical	CBS-All	CBS-unknown	CBS-4RT	CBS-AD	IDT	PD**
**MoCA - n, mean, (SD)**	76<br>26.8†<br>(2.2)	96<br>21.9<br>(4.7)	51<br>22.5ϰ<br>(3.6)	22<br>23.1ϰ<br>(5.0)	23<br>19.7<br>(6.0)	39<br>20.4<br>(7.4)	23<br>21.8ϕ<br>(6.1)	9<br>22.9ϕ<br>(5.3)	7<br>12.4<br>(9.0)	24<br>21.9<br>(6.5)	1833<br>24.9*<br>(3.6)
**ECAS - n, mean, (SD)**	76<br>116.3†<br>(10.6)	86<br>88.7<br>(22.1)	46<br>89.4ϰ<br>(17.8)	21<br>97.0ϰ<br>(22.0)	19<br>78.3<br>(27.8)	27<br>84.3<br>(33.0)	14<br>96.7ϕ<br>(24.9)	7<br>88.1<br>(26.6)	6<br>55.0<br>(39.0)	22<br>95.3<br>(18.8)	-
**ACE-III - n, mean, (SD)**	76<br>95.1†<br>(5.3)	91<br>78.8Ω<br>(13.1)	50<br>80.5ϰ<br>(9.5)	20<br>84.0ϰ<br>(9.8)	21<br>69.8<br>(18.6)	31<br>71.9<br>(23.5)	17<br>76.3ϕ<br>(20.1)	7<br>78.1<br>(17.0)	7<br>54.3<br>(31.2)	25<br>80.7<br>(12.1)	-
**Attention - mean, (SD)**	17.3*<br>(1.2)	15.8Ω<br>(2.3)	15.8<br>(2.1)	16.7ϰ<br>(1.9)	14.9<br>(2.3)	14.3<br>(4.1)	14.8ϕ<br>(3.2)	16.1ϕ<br>(2.5)	10.8<br>(5.7)	16.8<br>(2.1)	-
**Memory - mean, (SD)**	24.4†<br>(2.6)	21.0Ω<br>(4.6)	21.6ϰ<br>(3.5)	21.8<br>(4.4)	18.5<br>(6.4)	18.4<br>(7.3)	19.6<br>(7.5)	20.0ϕ<br>(4.9)	13.7<br>(5.8)	19.5<br>(4.1)	-
**Fluency - mean, (SD)**	12.3†<br>(1.6)	6.4<br>(3.4)	6.3<br>(3.0)	7.9ϰ<br>(3.2)	5.3<br>(3.9)	7.3<br>(4.1)	8.4<br>(3.4)	6.5<br>(4.4)	5.7<br>(5.2)	7.6<br>(4.1)	-
**Language - mean, (SD)**	25.7†<br>(0.6)	23.4Ω<br>(3.8)	23.9ϰ<br>(2.3)	24.8ϰ<br>(1.5)	20.5<br>(6.6)	21.5<br>(6.0)	22.5ϕ<br>(4.7)	23.6ϕ<br>(2.3)	16.6<br>(8.1)	23.6<br>(3.4)	-
**Visuospatial - mean, (SD)**	15.4†<br>(1.0)	12.2Ω<br>(3.1)	12.6ϰ<br>(2.5)	12.9ϰ<br>(2.9)	10.4<br>(3.8)	10.4<br>(4.9)	11.0<br>(4.5)	11.9<br>(5.4)	7.4<br>(5.7)	13.5<br>(2.3)	-

Cognitive screening measures in PROSPECT study natural history cohort. Addenbrooke's cognitive examination 3: ACE-III, Edinburgh cognitive and behavioural ALS screen: ECAS, Indeterminate: IDT, PSP-Richardson syndrome: PSP-RS, Montreal cognitive assessment: MoCA, CBS-Alzheimer's disease: CBS-AD, CBS-4 repeat tau: CBS-4RT, Parkinson's disease: PD, standard deviation: SD. Group and sub-group comparisons made with logistic regression analyses that used gender, age at symptom onset and disease duration at testing as covariates - **†** = FDR adjusted P<0.05 vs. all disease groups, ***** = FDR adjusted P<0.05 vs. PSP All and CBS All, **Ω** = FDR adjusted P<0.05 vs. CBS All, **ϕ** = FDR adjusted P<0.05 vs. CBS-AD, **ϰ** = FDR adjusted P<0.05 vs. PSP-Cortical, **** =** data from the Tracking Parkinson's study.

© 2019 Jabbari E et al. *JAMA Neurology*.

eTable 4. Genetic Analyses in the PROSPECT Study Natural History Cohort

	Controls*	PSP-All	PSP-RS	PSP-Subcortical	PSP-Cortical	CBS-All	CBS-unknown	CBS-4RT	CBS-AD	IDT	PD**
**No. of cases genotyped**	-	81	42	20	19	33	20	6	7	20	1566
***MAPT* H1/H1 frequency (%)**	67.1	88.9	90.5	85.0	89.5	78.8	80.0	83.3	71.4	75.0	67.2**‡**
***APOE*-ε4 allele frequency (%)**	15.1	6.8	9.5	2.5	5.3	12.1	7.5	0 **ϕ**	35.7	10.0	12.8**Ω**
***TRIM11* (rs564309) MAF (%)**	9.6	9.3	7.1	15.0	7.9	12.1	10.0	33.3**ϕ**	0	25.0	14.7

Genetic analyses in the PROSPECT study natural history cohort. Apolipoprotein E: *APOE*, Indeterminate: IDT, PSP Richardson syndrome: PSP-RS, minor allele frequency: MAF, microtubule associated protein tau: *MAPT*, CBS-Alzheimer's disease: CBS-AD, CBS-4 repeat tau: CBS-4RT, Parkinson's disease: PD, tripartite motif-containing protein 11: *TRIM11*. Group and sub-group comparisons of genetic data made with Fisher's exact test - **‡** = Bonferroni corrected P<0.05 vs. PSP-All and CBS-All, **Ω** = Bonferroni corrected P<0.05 vs. PSP-All, **ϕ** = Bonferroni corrected P<0.05 vs. CBS-AD. ***** = White control frequencies derived from dbSNP, ****** = data from the Tracking Parkinson's study.

© 2019 Jabbari E et al. *JAMA Neurology*.

eTable 5. Volumetric MRI Measures in the PROSPECT Study Natural History Cohort

	Controls	PSP-All	PSP-RS	PSP-Subcortical	PSP-Cortical	CBS-All	CBS-unknown	CBS-4RT	CBS-AD	IDT	p-value
n	35	44	25	11	8	17	7	5	5	12	
Pons-Midbrain ratio	2.45 (0.22)	2.60* (0.24)	2.61* (0.23)	2.61 (0.22)	2.55 (0.23)	2.48 (0.23)	2.57 (0.22)	2.40 (0.22)	2.44 (0.22)	2.52 (0.22)	$2.46e^{-9}$
Pons	14.72 (1.70)	13.25** (1.76)	13.06** (1.71)	13.70 (1.70)	13.17* (1.75)	13.67* (1.68)	14.09 (1.67)	13.73 (1.66)	13.57 (1.66)	14.55 (1.66)	$2.73e^{-9}$
Midbrain	5.99 (0.53)	5.09** (0.54)	5.01** (0.54)	5.23** (0.54)	5.16** (0.55)	5.54* (0.52)	5.54 (0.52)	5.73 (0.52)	5.55 (0.52)	5.77 (0.52)	$5.99e^{-7}$
Medulla	4.47 (0.43)	3.99** (0.44)	3.94** (0.43)	4.09* (0.43)	4.03* (0.44)	4.27 (0.42)	4.38 (0.42)	4.16 (0.42)	4.41 (0.42)	4.29 (0.42)	0.003
Cerebellum	103.10 (9.84)	99.95 (10.35)	102.24 (9.86)	102.24 (9.86)	94.88* (10.18)	99.70 (9.91)	101.47 (9.66)	98.33 (9.62)	101.77 (9.61)	103.72 (9.63)	$1.96e^{-4}$
Frontal Lobe	155.76 (11.12)	144.06** (11.78)	150.79 (11.14)	150.79 (11.14)	133.94** (11.50)	137.95** (11.28)	141.90* (10.92)	138.12** (10.88)	135.24** (10.87)	144.94* (10.89)	$1.25e^{-5}$

© 2019 Jabbari E et al. *JAMA Neurology*.

**Parietal Lobe**	103.75 (9.22)	99.14* (10.38)	102.26 (9.23)	102.26 (9.23)	90.10** (9.53)	86.73** (9.94)	95.29 (9.05)	76.99** (9.02)	88.34** (9.01)	97.47* (9.02)	$3.89e^{-8}$
**Temporal Lobe**	115.03 (10.12)	106.83** (10.67)	109.23 (10.13)	109.23 (10.13)	99.87** (10.46)	100.79** (10.22)	109.60 (9.93)	95.18** (9.89)	99.89* (9.88)	106.91* (9.90)	$1.98e^{-4}$
**Occipital Lobe**	47.30 (4.87)	45.58 (5.19)	46.49 (4.88)	46.49 (4.88)	41.97* (5.03)	43.34* (4.97)	48.29 (4.78)	40.28** (4.76)	42.09* (4.76)	42.63* (4.76)	0.003
**Central Structures**	41.56 (3.56)	36.33** (3.56)	36.35** (3.57)	36.35** (3.57)	35.01** (3.69)	36.54** (3.41)	37.07* (3.50)	37.42* (3.49)	36.96* (3.48)	38.81* (3.49)	$1.42e^{-5}$
**Ventricles**	35.80 (19.25)	48.76* (20.42)	39.56 (19.28)	39.56 (19.28)	57.01* (19.91)	59.83** (19.56)	44.93 (18.90)	60.81* (18.83)	75.75** (18.81)	48.53* (18.84)	$2.25e^{-4}$

Volumetric MRI measures in the PROSPECT study natural history cohort. Indeterminate: IDT, PSP-Richardson syndrome: PSP-RS, CBS-Alzheimer's disease: CBS-AD, CBS-4 repeat tau: CBS-4RT. Marginal mean volumes in ml (and standard deviation). Final column p-value of the overall F-test from between subjects design including total intracranial volume, age at scan and sex. Other within-cell p-values are FDR adjusted. ** = $P<0.001$, * = $P<0.05$ from t-test comparing each group to controls.

© 2019 Jabbari E et al. *JAMA Neurology*.

eMethods. Fluid Biomarkers, Genetics, and Neuroimaging

Fluid biomarkers: We processed non-fasting blood and CSF samples using standardised protocols and stored 0.5ml aliquots at -80°C within 60 minutes of sample collection. We measured serum NF-L using an ultrasensitive single molecule array (Simoa) assay.[1] In addition, where available, CSF samples from cases were tested for total tau (T-tau) and Aβ1-42 levels (INNOTEST ELISA – Fujirebio Europe N.V., Gent, Belgium).[2] A subset of PD cases from the Tracking Parkinson's study underwent baseline serum NF-L testing using the Simoa assay.

Genetics: DNA was extracted from blood samples of PSP, CBS, IDT and PD cases. DNA samples subsequently underwent genotyping at UCL Institute of Child Health using the Illumina NeuroChip[3] for white PROSPECT cases and the Illumina Human Core Exome array[4] for white Tracking Parkinson's cases. Standard steps taken for data quality control and single nucleotide polymorphism imputation, as previously described.[5] White control allele/haplotype frequencies were derived from dbSNP (www.ncbi.nlm.nih.gov/snp). *MAPT* H1/H1 (determined by rs1800547 genotype), *TRIM11* (determined by rs564309 minor allele frequency) and *APOE*-ε4 allele (determined by rs429358 and rs7412 genotypes) frequencies were calculated using our imputed genetic datasets.

Neuroimaging: Scan protocols were designed at the outset of the study to closely match across centres, based on the international Genetic Frontotemporal Dementia Initiative protocols (MP-RAGE, TR 2s, TE 2.93ms, Flip angle 8deg, 1.1mm isotropic).[6] T1-weighted images were processed using the recon-all pipeline with brainstem structures of FreeSurfer v6.0.0 (surfer.nmr.mgh.harvard.edu) into subcortical segments and cortical surface parcellations. Regional analyses were performed using volume measures from 68 Desikan-Killiany atlas cortical regions and 38 subcortical volume measures from the segmentation.[7,8] We combined volume measures from the parcellation to calculate cortical grey matter volumes of the frontal, temporal, parietal, occipital lobes. Hippocampi and amygdalae volumes are included in the temporal lobe data. The remaining segmentation regions were combined into central structures (basal ganglia, thalamus, accumbens), cerebellar grey matter, brainstem, and ventricles. The images were additionally segmented into grey, white and CSF modulated probability maps using CAT12 (neuro.uni-jena.de/cat) in order to obtain total intracranial volume (TIV) measures. We also calculated the ratio of the pons to midbrain volume.

© 2019 Jabbari E et al. *JAMA Neurology*.

eFigure. Serum NF-L ROC Curve for APS vs PD

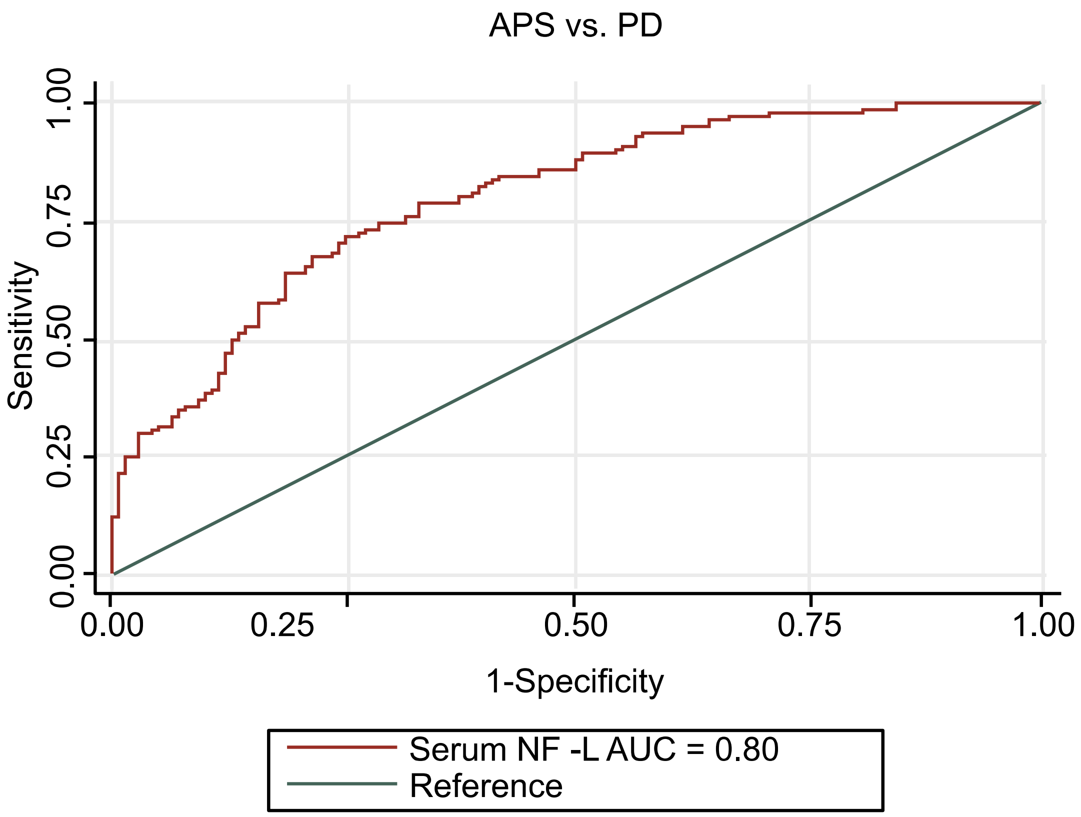

Receiver operating characteristic (ROC) curve for distinguishing APS from PD using serum NF-L. Atypical parkinsonian syndrome (consisting of all PSP and CBS cases): APS, Parkinson's disease: PD, neurofilament light chain: NF-L, area under the curve: AUC.

© 2019 Jabbari E et al. *JAMA Neurology*.

eReferences:

1. Kuhle J, Barro C, Andreasson U, et al. Comparison of three analytical platforms for quantification of the neurofilament light chain in blood samples: ELISA, electrochemiluminescence immunoassay and Simoa. *Clin Chem Lab Med* 2016; 54(10): 1655-1661.

2. Faull M, Ching SY, Jarmolowicz AI, Beilby J, Panegyres PK. Comparison of two methods for the analysis of CSF Aβ and tau in the diagnosis of Alzheimer's disease. *Am J Neurodegener Dis* 2014; 3(3): 143-151.

3. Blauwendraat C, Faghri F, Pihlstrom L, et al. NeuroChip, an updated version of the NeuroX genotyping platform to rapidly screen for variants associated with neurological diseases. *Neurobiol Aging* 2017; 57: e9-e247.

4. Guo Y, He J, Zhao S, et al. Illumina human exome genotyping array clustering and quality control. *Nat Protoc* 2014; 9: 2643-62.

5. Reed E, Nunez S, Kulp D, Qian J, Reilly MP, Foulkes AS. A guide to genome-wide association analysis and post-analytic interrogation. *Stat Med* 2015; 34: 3769-92.

6. Rohrer JD, Nicholas JM, Cash DM, van Swieten J, Dopper E, Jiskoot L, et al. Presymptomatic cognitive and neuroanatomical changes in genetic frontotemporal dementia in the Genetic Frontotemporal dementia Initiative (GENFI) study: a cross-sectional analysis. *Lancet Neurol* 2015; 14(3): 253-262.

7. Fischl B, Salat DH, Busa E, et al. Whole brain segmentation: automated labeling of neuroanatomical structures in the human brain. *Neuron* 2002; 33(3): 341-355.

8. Iglesias JE, Van Leemput K, Bhatt P, et al. Bayesian segmentation of brainstem structures in MRI. *Neuroimage* 2015; 113: 184-195.

© 2019 Jabbari E et al. *JAMA Neurology*.
